# Muskie Lunacy: Does the Lunar Cycle Influence Angler Catch of Muskellunge (*Esox masquinongy*)?

**DOI:** 10.1371/journal.pone.0098046

**Published:** 2014-05-28

**Authors:** Mark R. Vinson, Ted R. Angradi

**Affiliations:** 1 U.S. Geological Survey, Great Lakes Science Center, Lake Superior Biological Station, Ashland, Wisconsin, United States of America; 2 U.S. Environmental Protection Agency, Office of Research and Development, National Health and Environmental Effects Research Laboratory, Mid-Continent Ecology Division, Duluth, Minnesota, United States of America; University of Texas Southwestern Medical Center, United States of America

## Abstract

We analyzed angling catch records for 341,959 muskellunge (*Esox masquinongy*) from North America to test for a cyclic lunar influence on the catch. Using periodic regression, we showed that the number caught was strongly related to the 29-day lunar cycle, and the effect was consistent across most fisheries. More muskellunge were caught around the full and new moon than at other times. At night, more muskellunge were caught around the full moon than the new moon. The predicted maximum relative effect was ≈5% overall. Anglers fishing exclusively on the peak lunar day would, on average, catch 5% more muskellunge than anglers fishing on random days. On some lakes and at night, the maximum relative effect was higher. We obtained angler effort data for Wisconsin, Mille Lacs (MN), and Lake Vermilion (MN). For Lake Vermilion there was a significant effect of the lunar cycle on angler effort. We could therefore not conclude that the lunar effect on catch was due to an effect on fish behavior alone. Several factors affected the amount of variation explained by the lunar cycle. The lunar effect was stronger for larger muskellunge (>102 cm) than for smaller fish, stronger in midsummer than in June or October, and stronger for fish caught at high latitudes (>48°N) than for fish caught further south. There was no difference in the lunar effect between expert and novice muskellunge anglers. We argue that this variation is evidence that the effect of the lunar cycle on catch is mediated by biological factors and is not due solely to angler effort and reflects lunar synchronization in feeding. This effect has been attributed to variation among moon phases in lunar illumination, but our results do not support that hypothesis for angler-caught muskellunge.

## Introduction

Studies of the effects of the moon on fish behavior have shown that fish spawning [1]–[4], vertical and horizontal movement [5], [6], migration [7], [8], activity [9], feeding [10], [11], and vulnerability to commercial [12], [13] or recreational fishing [14]–[16] may be synchronized to lunar cycles. The idea that fish-feeding behavior is related to the phases of the moon was popularized, if not quite quantified, by John Alden Knight in 1936 with the publication of his “Solunar” tables [17]. Solunar tables predict days and hours of increased fish and wildlife activity−times when the likelihood of fishing and hunting success is increased. The Solunar effect is due in part to the influences of the sun and moon, which are greatest when they are aligned, as at the time of the full and new moon.

The muskellunge (*Esox masquinongy*) is arguably the premier non-salmonid freshwater game fish in North America. The species supports a sport fishery worth billions of dollars to the economies of the United States and Canada [18], [19]. Because it grows to a large size (documented to 30 kg and 140 cm [20]), fights hard, and is notoriously difficult to catch, there is a mystique, even a nobility to the species [21]. Accordingly, lore has accumulated around muskellunge behavior, a central theme of which is the role of moon phases on the species' feeding activity and catchability.

Anglers' beliefs about lunar-cycle effects on game fish feeding and catchability are sometimes corroborated by scientific study [22], [23]. Catch per unit of effort (CPUE) of pike (*Esox lucius*) in a German lake was elevated during the full and new moon [16]. The effect of the moon phase on CPUE was less than the effect of time of day, fishing pressure, and water temperature, however. Donabauer [23], using angler-reported data, found that more trophy pike in the state of Indiana were caught around the full and new moons than otherwise. Landsman used implanted accelerometers to track the activity of muskellunge in an Ontario river [24]. He found that although muskellunge were inactive >70% of the time, there was some evidence of a lunar effect on activity.

Much information related to the influence of the moon on muskellunge feeding behavior and catchability has appeared in popular literature. Bucher [25] claimed that:

*“…my [fishing] logs revealed the predictable frequency of big fish catches during the peak moon phases of full moon and new moon. Specifically, a lot more big muskellunge… were taken right on the actual scheduled calendar day of both the full or new (dark) moon peak and continued for a three to five day stretch afterwards”*

Dettloff [26] examined catch records from the Chippewa Flowage, Wisconsin, and concluded that:

*“[T]he best 30 pound muskellunge day of the entire lunar month is the day after the new moon, [when] the odds of catching a big fish are twice as good as they are on the average lunar day”*

Heting [27] examined his catch records and discovered that:

*“An average moon period should account for 25 percent of the 40-inch and better muskies in my boat during the past five years. The full moon period produced the most muskies in my sample and accounted for 34.7 percent of my total of fish over 40 inches”*

Although they are based on, at most, a few thousand catch records, these conclusions reflect the experience of expert anglers (including professional fishing guides) and should not be discounted for lack of peer-review [28]. We tested if these assertions were supported by analysis of a much larger dataset of angler-reported muskellunge catches. Our objectives were to 1) test for a lunar effect on angler-reported muskellunge catches, and 2) explore sources of variation in the effect of the lunar cycle on the catch. A better understanding of lunar influence on muskellunge behavior in the wild is of interest to muskellunge anglers. We think our findings will also be of interest to chronobiologists, and will help fishery managers better understand aspects of muskellunge fisheries that have a behavioral dimension−both for anglers and for muskellunge.

## Materials and Methods

**Figure 1:**
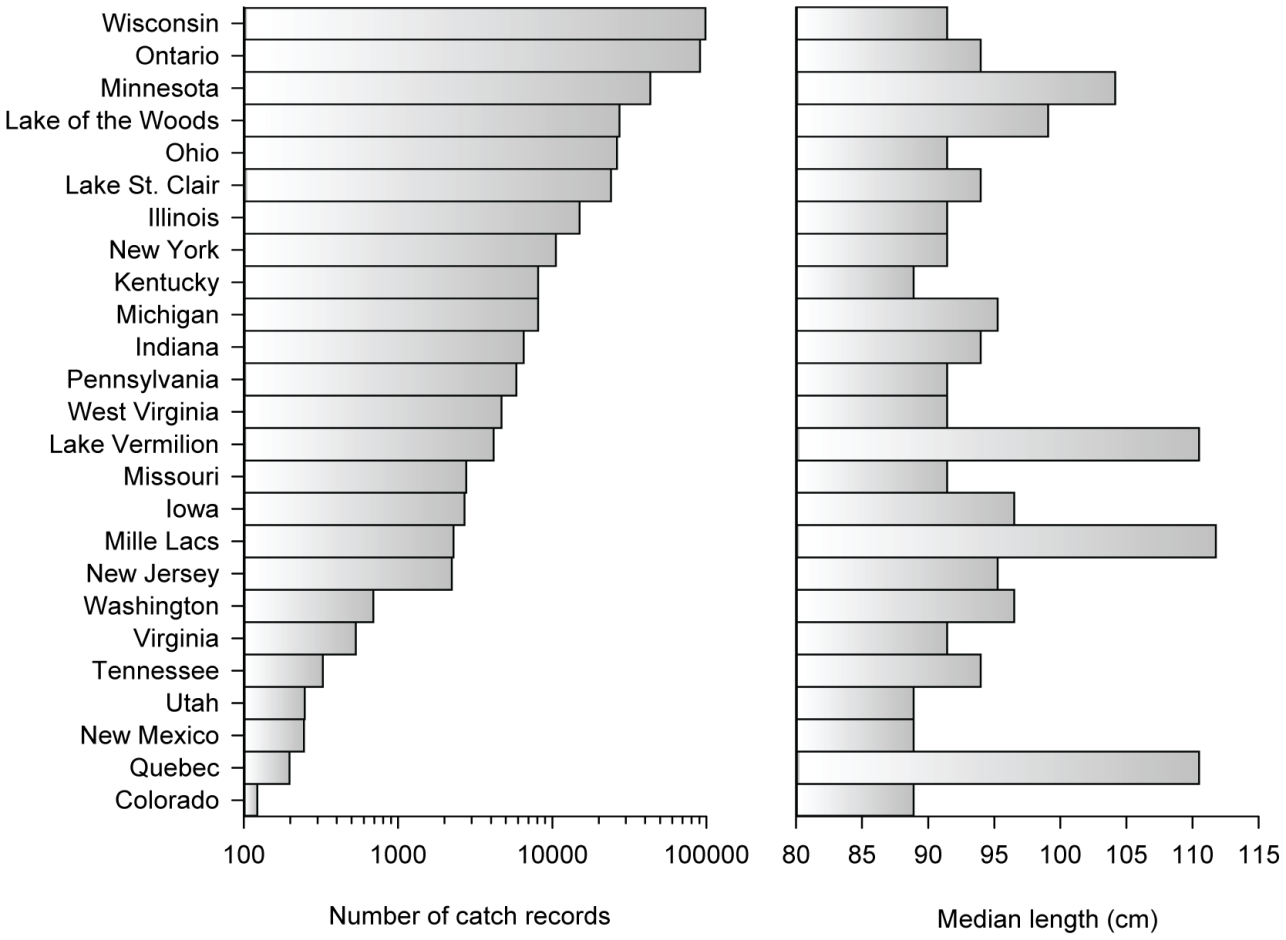
Number of catch records and median fish length by state, province, and water body. Groups with <100 fish are not shown.

We obtained 341,959 muskellunge catch records for 1970 to 2013 from Muskies Inc., a service oriented, non-profit organization (http://www.muskiesinc.org). Data were self-reported by anglers in the United States and Canada (Fig. 1, Fig. S1). The format of the data was a single record for each landed fish. Fields included, among others, angler name, date and hour caught, location caught (water body name and county), fish length, and whether or not the fish was released. We grouped records into two diel groups, eight geographic groups, and two “angler expertise” groups for analysis. Diel groups were daytime (0600–2100) and nighttime-caught muskellunge. Geographic groups included Minnesota, Wisconsin, Ohio, and Ontario, Lake of the Woods (MN and Ontario), and Lake St. Clair (MI and Ontario), and two fisheries for which we have creel data, Lake Vermilion (MN) and Mille Lacs (MN). Expertise groups included records for fish caught by anglers that caught the most fish: 87 anglers that each caught an average of 576.7 fish for a total of 50,171 fish, and anglers that caught the fewest fish: 7072 anglers that caught and average of 7.2 fish for a total of 50,802 fish (the rationale for the total number or records in each group is explained below). We do not know the amount of angling effort associated with each catch record. Over 99% of muskellunge were released, according to anglers.

From Department of Natural Resources creel survey data for Wisconsin (1993–2009), Lake Vermilion (2002–2009), and Mille Lacs (1999–2012) we extracted the number of hours anglers reported they spent targeting muskellunge and the number of muskellunge caught on each survey date. From these data we calculated the total angler hours and catch per unit of effort (number caught per hour) for each lunar day. To filter out implausible creel survey data, we excluded angler effort records for which the number of individual angler hours for the day was ≥16 or the number of anglers in the fishing party was >6. We only included anglers self-identifying as muskellunge anglers or who stated they were expending ≥50% of their effort targeting muskellunge.

For each catch and creel survey record, we determined the number of lunar cycles between a known full moon date (January 5, 1901) from U.S. Navy moon phase tabulations (http://aa.usno.navy.mil/data/docs/MoonPhase.php) and the catch or creel survey date. We then subtracted the number of complete lunar cycles since the known full moon date leaving a fraction of one 29.530588-day synodic cycle, which we converted to lunar day and rounded to a whole number from 1 to 29. We back- checked our derived lunar days with the Navy tables to confirm the reliability of our method.

**Figure 2:**
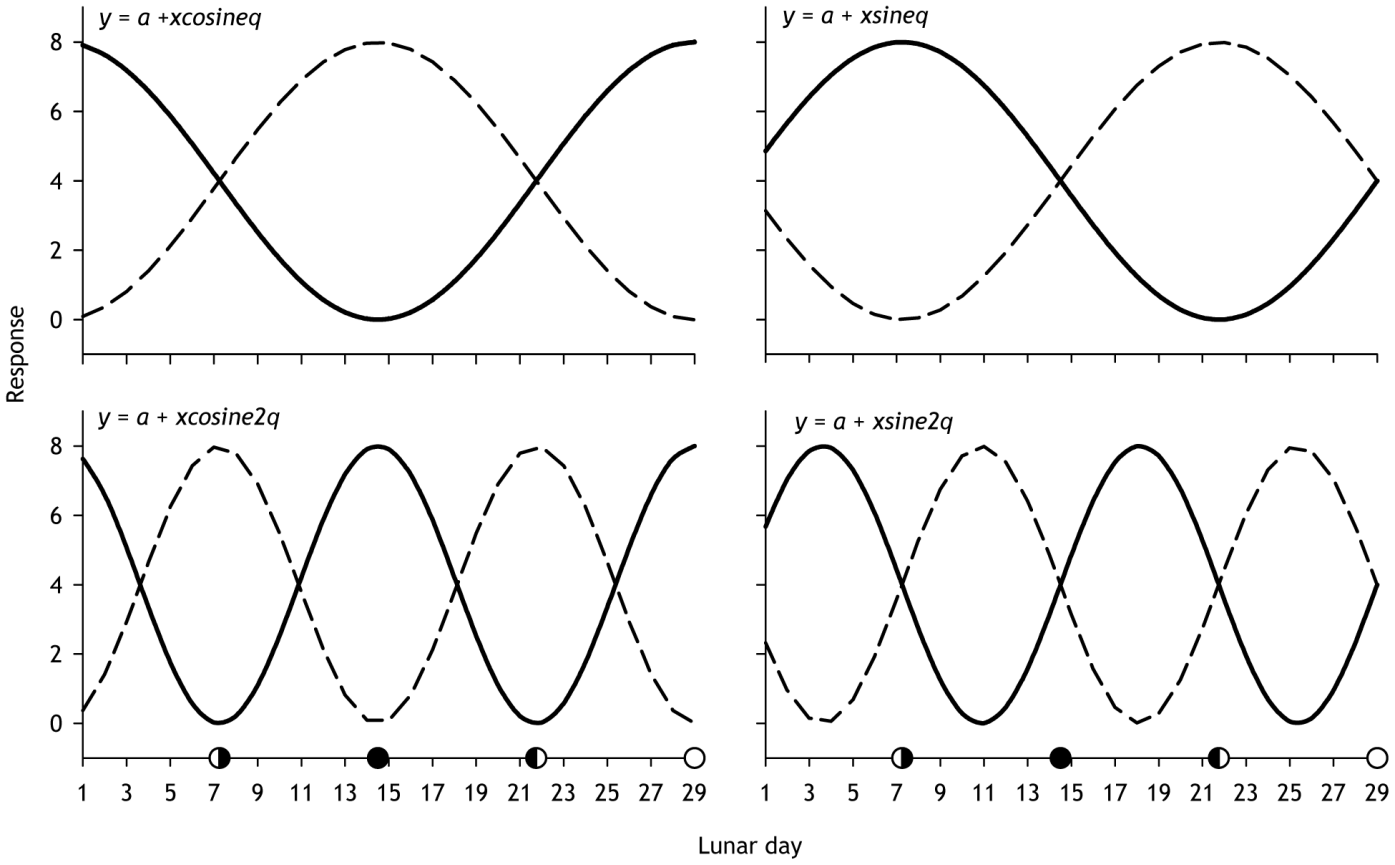
Simulated patterns for single lunar predictor periodic regression models. For each simulation, *a* was set at 4. For models with solid lines *x* was set at 4. For models with dashed lines *x* set at -4. The lunar cycle starts at 0001 h on the morning after the full moon. Response corresponds to catch, CPUE, or angler effort. In these simulations, the maximum relative effect is 100%.

We tested for a lunar cycle effect on the number of angler caught muskellunge, hours of angling effort (from creel survey data), and catch per unit effort (from creel survey data) using periodic regression [29]. The 29-day lunar cycle was divided into 360° (or 2π radians) to give each lunar day an angular equivalent, *θ* (theta). We square root transformed catch data (counts of muskellunge caught on a given day) to produce a normally distributed data set [30]. We used least squares regression (SAS for Window v.9.2; SAS Institute Inc.) using four single-predictor response models (Fig. 2) to test for lunar and semi-lunar cycles in catch or effort.We determined the best single predictor model using the Akaike Information Criterion [31]. We also determined the best two-predictor model for catch, angling effort, and CPUE. Adding a second predictor allows models to be fit to asymmetric patterns (e.g., the full moon influence is greater than the new moon influence) or to patterns offset from a simple lunar and semi-lunar cycles (Fig. S2). Additional predictors may improve model fit (Fig. S3), but the improvement is likely to be spurious or difficult to interpret [29]. For each significant model, we calculated the maximum relative effect as the model-predicted percent increase in the number of fish caught when fishing on the peak day when the predicted lunar effect is maximum, over fishing on completely random days ( = mean catch). Assuming no effort bias, the maximum relative effect is the maximum percent reduction in the time needed to catch each muskellunge attributable to a lunar effect on catchability. Normal probability plots and residual plots showed that least squares regression assumptions were met (Fig. S4). To reduce the likelihood of a type I error (false positive for regression *F*-test), we used the Šidák correction to control familywise error rate [32]. The periodic regression approach is optimal when response curves are sinusoidal [19]; otherwise, this approach may misrepresent the true shape of the response. We therefore fit local regressions to the catch data (LOESS, SigmaPlot for Windows v. 12.5, Systat Software Inc.).

Preliminary analysis revealed an effect of sample size on the strength of periodic regressions. To examine sources of variation in the strength of models while controlling for the sample size effect, we used random sampling without replacement to extract 20,000- or 30,000-record subsets from the data 100 times for each factor level. We ran the overall best fitting single-predictor regression model for all 100 replicate subsets and used the mean *R^2^* value to examine the effect of the factor on the lunar effect. Factors included latitude, month, and fish length. We determined latitude for the location of the lake for Canadian records and for the centroid of the named county for U.S. records.

Preliminary analysis also revealed a strong effect of day of the week on the number of fish caught (Fig. S5). The most fish were caught on Saturdays (23% of total) and the fewest from Monday−Thursday (each day ≈11% of the total). The pattern of angler effort by day of the week from creel survey data was identical to catch. However, there was no effect of the lunar cycle on the frequency of occurrence of any day of the week (*R*<0.001 in every case). In other words, for the period of record, there were not more or fewer full or new moons on weekend days than on weekdays. Because of the large latitudinal range in catch records (34–53°, Fig. S1), there was an inherent seasonal bias in the records. At lower latitudes, a high percentage of the catch was during winter and early spring when high-latitude water bodies are ice covered (Fig. S6). We therefore restricted our analysis to fish caught from June to October unless otherwise specified.

## Results

### Lunar Effect on Catch

The effect of the lunar cycle on daytime muskellunge catch was significant (*P*<0.05; Table 1) for all geographic groupings except Wisconsin (*P* = 0.23), Ohio (*P* = 0.16), and Mille Lacs (*P* = 0.06). By the more conservative familywise significance threshold (*P*<0.005), the effect of the lunar cycle on catch in Minnesota was not statistically significant.

**Table 1:** Periodic regression results; *N* = 29 in all cases. Top catch is the catch of the most experienced anglers.

Dependent variable	Best predictor	Number of fish^a^ or hours^b^	*F*	*P* c	*R^2^*	Max. relative effect^d^ (%)	Best two predictors	*P*	*R^2^*	Max. relative effect (%)
Muskies Inc. catch data										
Daytime catch^e^	cosine2*θ*	228,172	37.4	<0.0001^e^	0.58	4.8	cosine2*θ*, −sine*θ*	<0.0001	0.59	4.8
Nighttime catch^f^	cosine*θ*	7708	18.6	0.0002	0.41	14.2	cosine2*θ*, cosine*θ*	<0.0001	0.74	28.2
Ontario catch	cosine2*θ*	73,421	81.5	<0.0001	0.75	10.1	cosine2*θ*, −cosine*θ*	<0.0001	0.77	11.6
Minnesota catch	cosine2*θ*	28,227	7.0	0.01	0.21	4.0	cosine2*θ*, cosine*θ*	0.03	0.24	5.5
Wisconsin catch	cosine2*θ*	70,371	1.5	0.23	0.05	Nd^g^	cosine2*θ*, cosine*θ*	0.37	0.07	Nd
Ohio catch	−sine2*θ*	13,124	4.6	0.16	0.15	Nd	−sine2*θ*, −cosine*θ*	0.07	0.18	ND
Lake of the Woods catch	cosine2*θ*	22,259	31.4	<0.0001	0.54	10.4	cosine2*θ*, cosine*θ*	<0.0001	0.63	14.9
Mille Lacs catch	cosine2*θ*	1262	3.8	0.06	0.12	Nd	cosine2*θ,* sine2*θ*	0.08	0.17	Nd
Lake Vermilion catch	cosine2*θ*	1771	25.6	<0.0001	0.49	19.1	cosine2*θ*, −sine2*θ*	0.0001	0.50	19.7
Lake St. Clair catch	cosine2*θ*	17,058	13.8	0.0009	0.34	14.0	cosine2*θ*, −cosine*θ*	<0.0001	0.59	26.4
High expertise catch^h^	cosine2*θ*	50,171	21.7	<0.0001	0.46	5.3	cosine2*θ*, −sine2*θ*	0.0001	0.50	5.6
Low expertise catch	cosine2*θ*	50,802	21.8	<0.0001	0.45	5.0	cosine2*θ*, −sine*θ*	0.0002	0.48	5.1
Department of Natural Resources creel data										
Wisconsin effort	−sine*θ*	91,095	1.4	0.25	0.05	Nd	cosine*θ*, −sine*θ*	0.32	0.08	Nd
Mille Lacs effort	cosine*2θ*	44,856	1.2	0.29	0.04	Nd	cosine2*θ*, sine*θ*	0.55	0.04	Nd
Lake Vermilion effort	cosine2*θ*	17,113	12.4	0.002	0.31	23.8	cosine2*θ*, cosine*θ*	0.001	0.40	36.0
Wisconsin CPUE	−sine2*θ*	1445	0.48	0.49	0.02	Nd	−sine2*θ*, sine*θ*	0.64	0.03	Nd
Mille Lacs CPUE	cosine2*θ*	358	2.2	0.15	0.07	Nd	cosine2*θ*, −cosine*θ*	0.17	0.13	Nd
Lake Vermilion CPUE	sine2*θ*	334	3.2	0.08	0.11	Nd	cosine2*θ*, cosine*θ*	0.12	0.15	Nd

a Catch and CPUE models.

b Effort models.

c The familywise significance rate (*α*) for 8 geographic group *F*-tests using the Muskies Inc. catch data is 0.006; the familywise significance rate for 6 tests using the creel data is 0.009.

d The percent increase in predicted number of muskellunge caught or predicted hours fished on the peak days relative to catch or fishing effort on random days. For catch, the maximum relative effect was calculated using back-transformed predicted catch.

e All catch models except “Night” excluded nighttime records and records from November – May.

f Includes all night records except records from November – May.

g Nd  =  not determined (regression *F* not significant).

h Excludes nighttime records and records from November – May.

**Figure 3:**
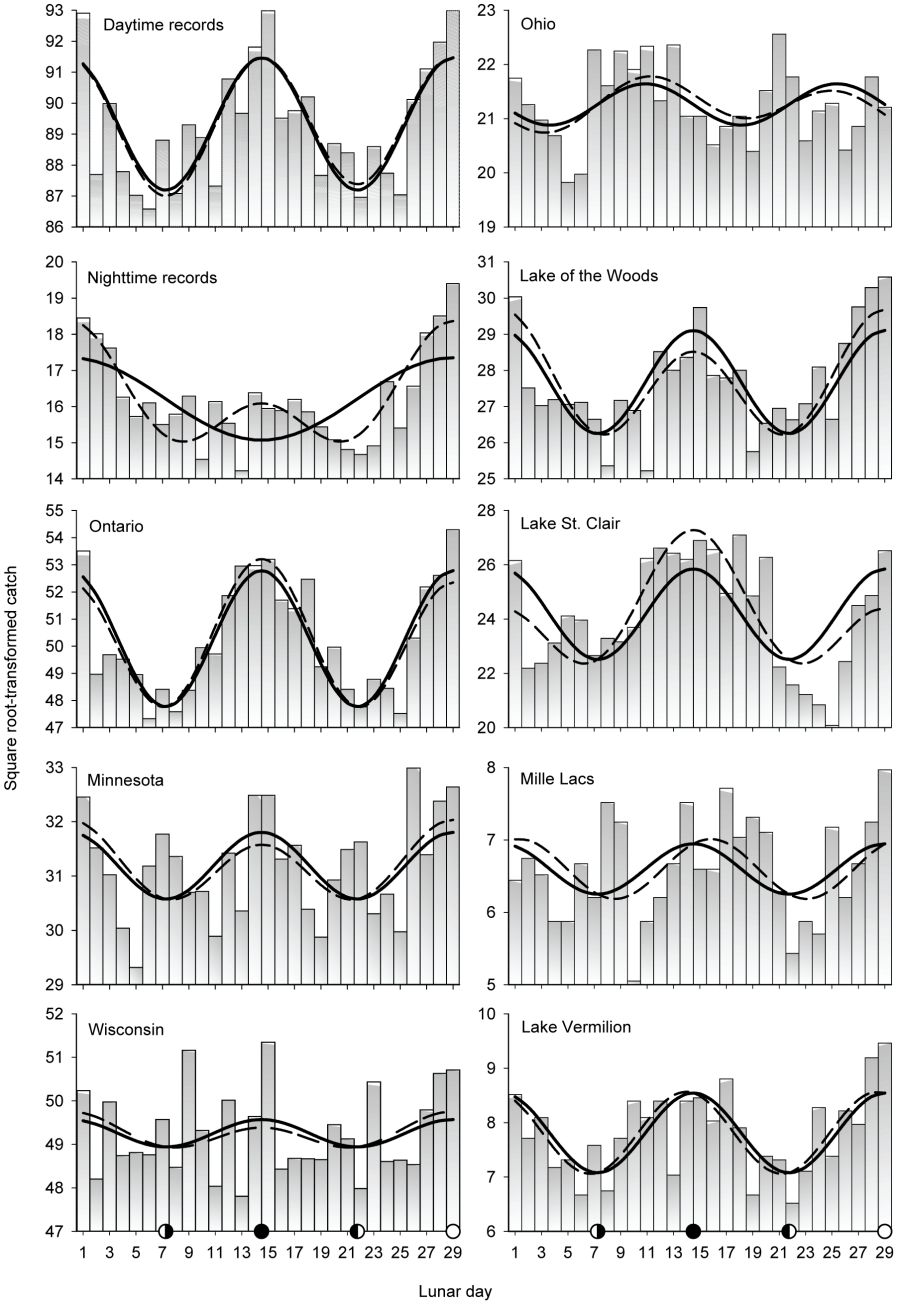
Periodic regression results for catch records. Bars are transformed counts. Solid regression lines are for the best single-predictor regression model. Dashed lines are for the best two-predictor model. Regression statistics given in Table 1.

Except for nighttime records, the best predictor of muskellunge catch was *cosine2θ* (where *θ*, theta, is the angular equivalent of lunar day 1–29), and the sign of the predictor was positive (Table 1, Fig. 3), meaning that more fish were predicted to be caught during the full and new moon periods that at other times (Fig 3). For nighttime records, the best predictor was *cosine2θ*, meaning that more fish were predicted to be caught during the full moon period than at other times.

Addition of a second predictor improved model fit in all cases but did not affect the pattern in most cases. Two exceptions were nighttime records and Lake St. Clair. Addition of the predictor *cosine2θ* to the nighttime model significantly improved the fit of the model and shifted predicted minimum catch from the new moon to the periods between the full and new moon. Addition of the predictor –*cosineθ* to the Lake St. Clair model increased the influence of the new moon on predicted catch over that of the full moon. For Minnesota, Wisconsin, and Lake of the Woods, the two-predictor model predicted slightly higher catch rates during the full moon than the new moon. For all daytime catch records combined, the pattern was symmetrical for the single and two-predictor models: the influence of the new and full moon on the catch was the same.

Alternative analysis of the catch data using local regression (LOESS) confirmed the two-predictor periodic regression results (Fig. S7) for daytime, nighttime, Ontario, Lake of the Woods, and Lake Vermilion catch. The local regression highlights complex patterns for Minnesota, Wisconsin, and Mille Lacs that were not adequately described by the fitted periodic models. In these cases, the lunar minima occurred during the quarter moon (e.g., near lunar day 4, 11, 18, and 26). This pattern was most apparent for Minnesota, where there were peaks in catch during the full, new, *and* half moons. At Lake St. Clair, decline in catch between new and full moon was less than the decline after the full moon.

The best two-predictor periodic regression models (*R* ^2^>0.63) were for Ontario, Lake of the Woods, and nighttime records (Table 1). Two-predictor models for all daytime records, Lake Vermilion, and Lake St. Clair were also strong (*R^2^*≥0.5). The maximum relative effect (the model-predicted percent increase in the number of fish caught when fishing on the peak day when the predicted lunar effect is maximum, over fishing on completely random days) was highest at night (28%) and at Lake St. Clair (26%). This means that anglers fishing at night exclusively on the peak lunar day (day 29, full moon) would catch 28% more muskellunge than anglers fish at night on random days. For all daytime records, the maximum relative effect of the lunar cycle was ≈5%. Therefore, although more muskellunge were caught during the day, the effect of the lunar cycle on the catch was greater at night.

### Angling Effort and Time Required to Catch a Muskellunge

**Figure 4:**
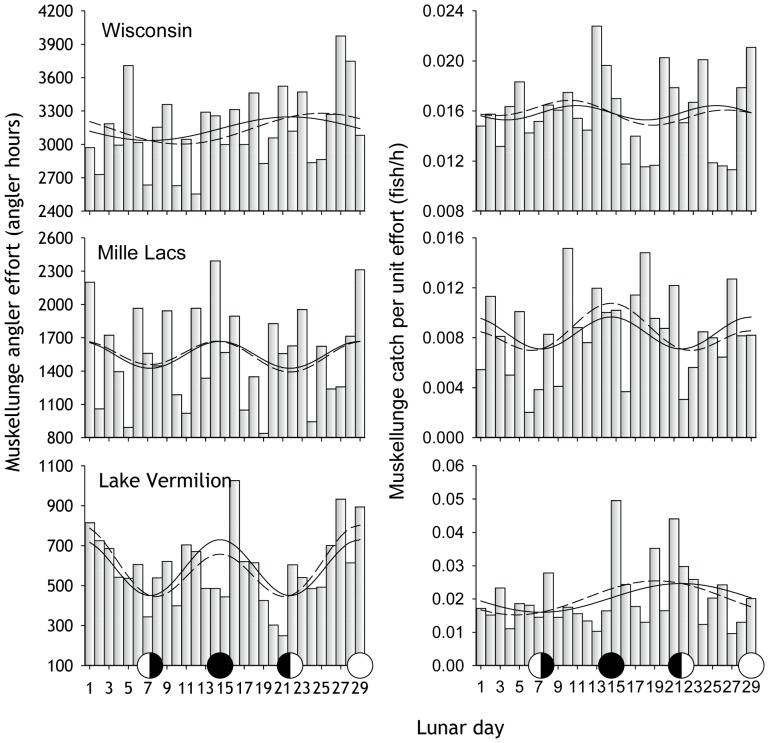
Periodic regression results for muskellunge fishing effort from creel surveys. Bars are Solid regression lines are for the best single-predictor model. Dashed regression lines are for the best two-predictor model. Regression statistics given in Table 1.

The effect of the lunar cycle on angler effort was not significant except at Vermilion Lake (Table 1) where effort was concentrated during the full and new moon periods (Fig. 4). The best model predicted that 36% more effort was expended on the predicted peak lunar day (day 29) than on the average day (Table 1). The lunar cycle did not explain significant variation in catch per unit of effort (CPUE) for any of the creel survey data sets (Table 1, Fig. 4).

Using published muskellunge CPUE estimates (Table 2) and the predicted maximum relative effect from our analysis (Table 1) we estimated the maximum decrease in the predicted number of hours required to catch a muskellunge (CPUE^−1^) when fishing only on predicted peak days relative to fishing on random days. The decrease in CPUE^−1^ was greatest for Lake of the Woods (5 h), and least for Ontario muskellunge fisheries (1.7 h).

**Table 2:** Reduction in the time required to catch a muskellunge based on catch per unit effort (CPUE) and the relative lunar effect based on the best two-predictor model (see Table 1).

Fishery	Max. relative effect (%)	CPUE (fish/h)	CPUE^−^(h/fish)	Reduction (h)	CPUE reference
Ontario	12	0.07	14	1.7	[45]
Minnesota	6	0.03	33	2.0	[46]
Lake of the Woods	15	0.03	33	5.0	[47]
Lake St. Clair	26	0.09	11	2.9	[48]

### Sample Size and the Lunar Effect

**Figure 5:**
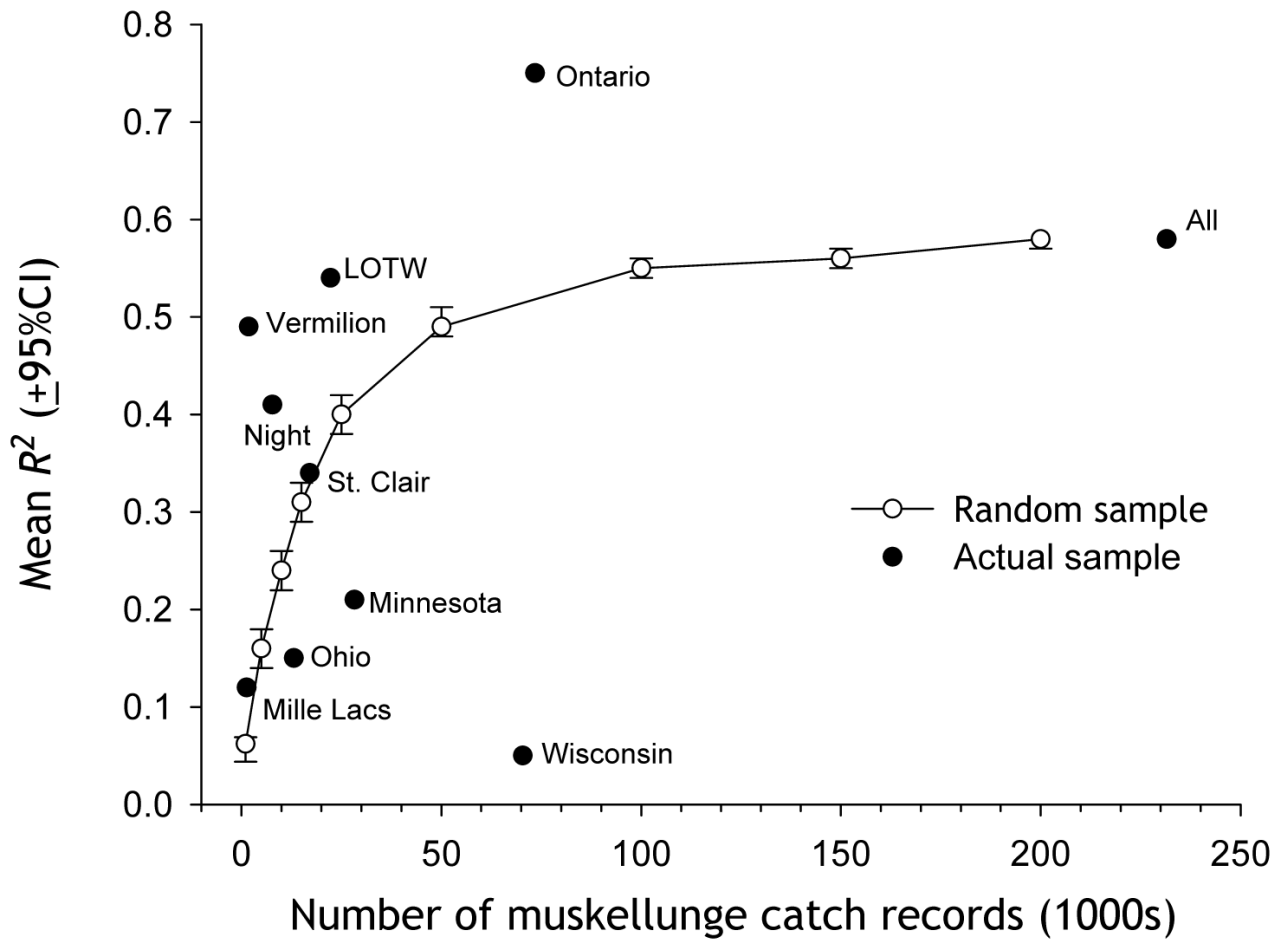
Sample size and the strength of the relationship between the lunar cycle and number of fish caught. For the random samples, *R^2^* based on models of the form *y = a+xcosine2θ*, where *y* =  number of fish caught and *θ* is the angular equivalent of lunar day. Actual model R^2^ values are from Table 1. LOTW  =  Lake of the Woods.

There was a relationship between sample size and the mean *R^2^* of models using *cosine2θ* to predict fish catch (Fig. 5). For random samples of 10,000 catch records, mean model *R^2^* was ≈0.24. A mean *R^2^* of 0.5, meaning that the lunar cycle explained half the overall variation in the catch, required a random sample of 50,000 records. Mean *R^2^* becomes nearly asymptotic near 0.6 after about 100,000 records. The relationship for Wisconsin was especially weak (*R^2^* = 0.05). Based on the number of catch records for Wisconsin, *R^2^* was expected to be ≈0.5. Ontario, and to a lesser extent, Lake of the Woods and Lake Vermilion, exhibited relationships considerably stronger than expected based on random sampling of the entire dataset.

### Month, Latitude, Fish Size, and Angler Expertise

**Figure 6:**
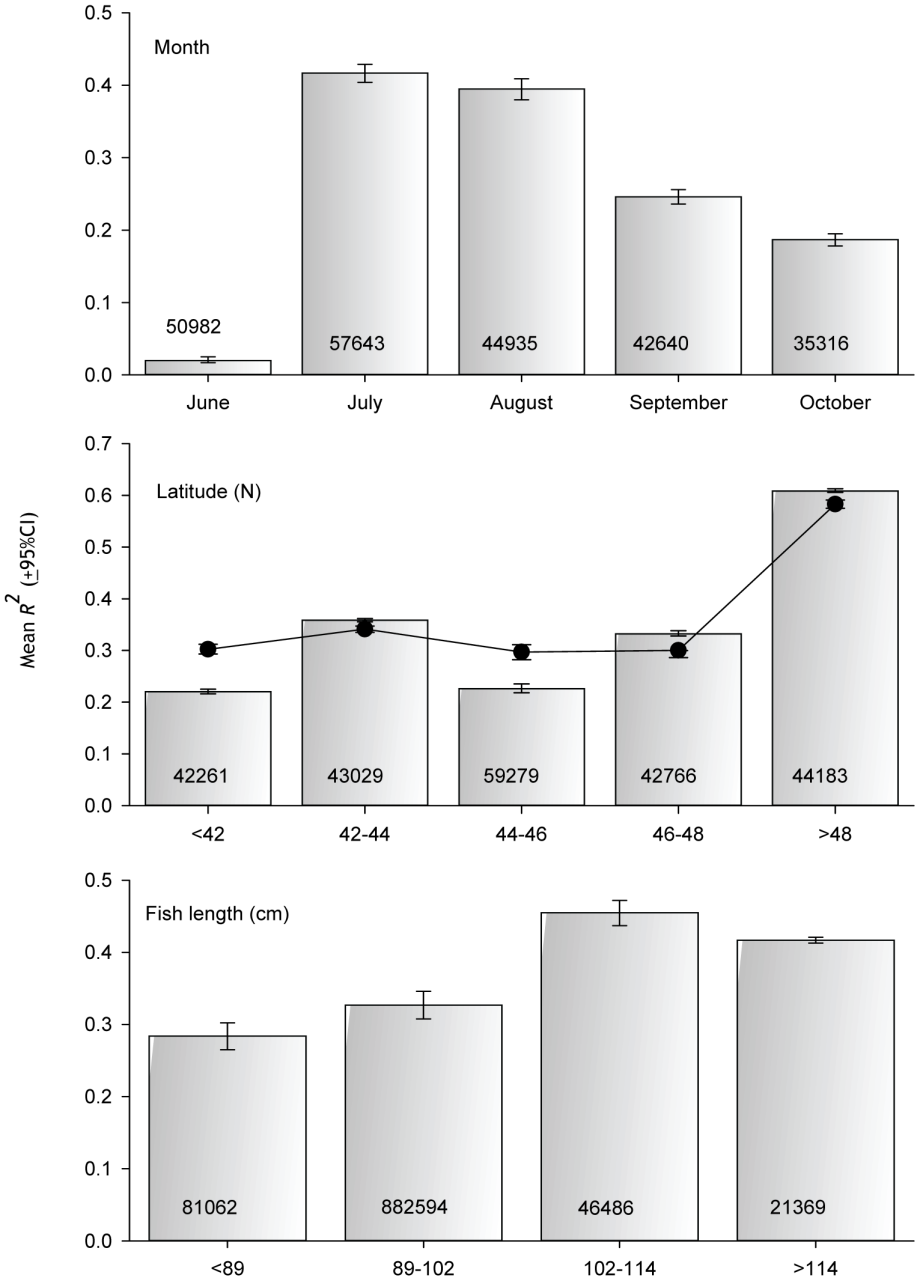
Factors influencing the strength of the relationship between the lunar cycle and number of fish caught. Mean *R^2^* values are for 100 model runs of 30,000 (latitude and month) or 20,000 (length) randomly selected daytime caught fish. All models are of the form *y = a+xcosine2θ*, where *y* =  number of fish caught and *θ* is the angular equivalent of lunar day. Value inside bars is N. Scatter plot for Latitude is the mean *R* ^2^ when June and October are excluded from regression models.

**Figure 7:**
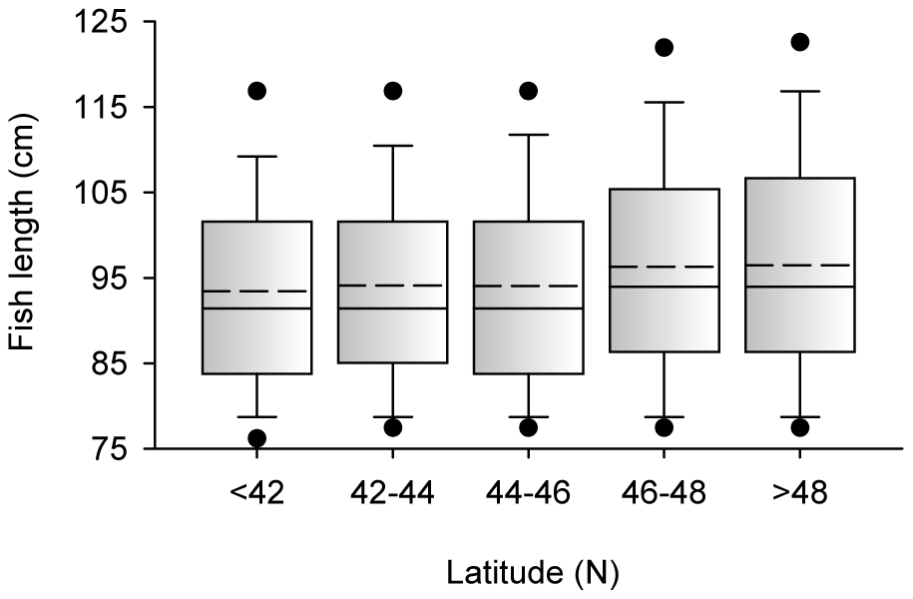
Length of fish by latitude for July to September. Dashed line is the mean; symbols are the 5^th^ and 95^th^ percentiles.

**Figure 8:**
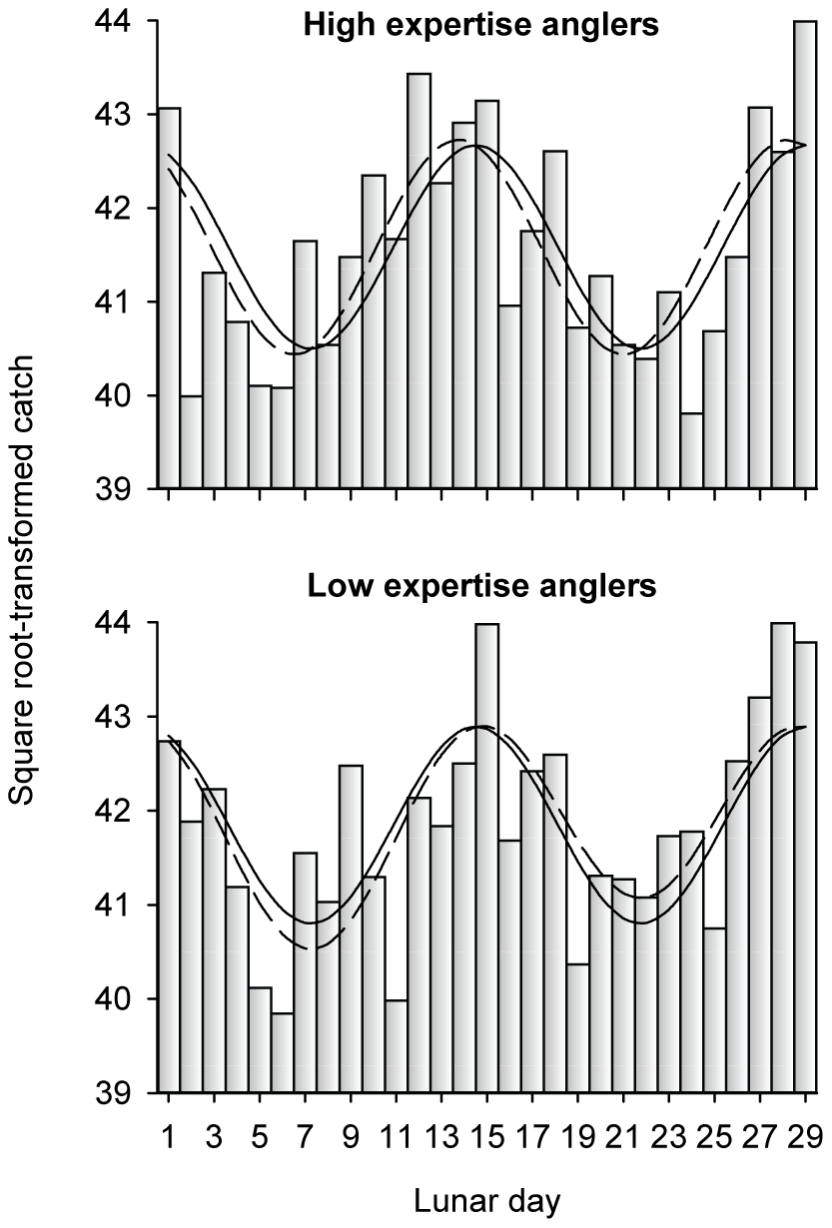
Periodic regression results for catch by high and low expertise anglers. Bars are transformed counts.

The relationship between the lunar cycle and catch (Fig. 6) was very weak in June (*R* ^2^ = 0.02) and strongest in July and August (*R^2^* = 0.4). The strength of the effect varied with latitude. For records from latitudes >48°N, the lunar cycle explained 60% of variation in catch (i.e., *R^2^* = 0.6; Fig. 6). For records from latitudes ≤48°N, the lunar cycle explained <36% of variation. To eliminate any seasonal bias remaining in the data (Fig. S6), we further restricted the data to only records from July – September (i.e., we removed June and October records). For this “summer only” subset, the effect of latitude was still present. High latitude summer-caught fish were larger than lower latitude fish (Fig. 7), but the mean effect was negligible, <3 cm. There was an effect of fish length on the strength of the lunar effect models (Fig. 6). Catch of fish <102 cm (≈40 inches) was less strongly related to the lunar cycle (*R^2^* = 0.33) than larger fish (*R^2^*≥0.42). There was no effect of angler expertise on the strength of the lunar effect models (Fig. 8). The lunar cycle explained about half the variation in the catch of ≈50,000 muskellunge caught by expert anglers and ≈50,000 muskellunge caught by anglers with low expertise (Table 1).

## Discussion

### Is There a Lunar Effect on Catch?

Sandell [33] summarized ≈67,000 muskellunge catch records using an earlier version of the same Muskies Inc. dataset we used. He concluded that the percentage of muskellunge caught during a 3 day new moon or a 3 day full moon period was not different from what would be expected of fish were caught randomly through the lunar cycle. He used a categorical analysis approach that is less sensitive for detecting a cyclic effect than periodic regression [29]. Landsman [24] felt that his data from muskellunge fitted with transmitters “lends credibility to lunar table predictions and behavioral observations made by anglers.” His data plots show that fish activity was highest during the waxing moon (about day 22 in our cycle, Fig. 2). The actual variation in activity through the lunar cycle was slight, amounting to a relative effect of <1%. Like us, he scaled the lunar cycle using the method of deBruyn and Meeuwig [29], but the waxing moon peak in activity he observed does not correspond to any that we observed. Fish swimming activity may not be a reliable indicator of feeding activity (and catchability) for a sit-and-wait predator like the muskellunge.

Our analysis of nearly 240,000 muskellunge catch records (all June – October catch records) provides prima facie evidence that catch rate varied with the lunar cycle. The strength of the lunar effect was lower than expected for Wisconsin. What, then, is different about Wisconsin relative to the other fisheries? The relatively small size of muskellunge caught in Wisconsin relative to fish caught in Minnesota, Lake of the Woods, Lake Vermilion, or Mille Lacs (but not Ontario) may be a factor (Fig. 1). As we have shown, catch of smaller fish was less influenced by the lunar cycle (Fig. 6). In Wisconsin, the catch was distributed across many more water bodies than in other geographic groups. The record for Ontario includes 211 water bodies from each of which 431 muskellunge were caught on average. In Minnesota, 338 fish were caught, on average, from each of 129 water bodies. In Ohio, 610 muskellunge were caught on average from each of 43 water bodies. In Wisconsin, only 153 fish were caught, on average, from each of 642 water bodies. If number of fish caught per water body is a reasonable proxy for water body area, then the Wisconsin muskellunge fishery is strongly skewed toward small lakes compared to the other major fisheries. This may account, in part, for the smaller average size of Wisconsin muskellunge (Fig. 1), but there may also be a link between water body size and the influence of the moon on muskellunge behavior. Another possibility relates to overall fishing pressure, which is discussed below.

In contrast to our findings based on angler-reported catches (the Muskies Inc. data), we found no lunar effect on muskellunge CPUE from creel surveys. The number of fish caught by anglers included in creel surveys was only a fraction (2%) of the number of catch reports in the fish in the Muskies Inc. data for the same water bodies, however. As we have shown, the absolute effect of the lunar cycle on catch is quite subtle and patterns are most evident when a large number of catch records is considered.

### Does Effort Bias Account for the Lunar Effect?

At Lake Vermilion, muskellunge-angling effort was concentrated on or about the full and new moons, matching the pattern of catch. For this reason, and because we have creel survey data for only a few fisheries, we cannot conclude that catch and effort are independent −that catch rates reflect *only* fish behavior since even if fish catchability were constant during the lunar cycle, more fish would be caught during the full and new moons as a consequence of increased angler effort at those times.

As for the lunar effect on catch, there is anecdotal evidence that muskellunge anglers concentrate their angling effort during what they perceive to be favorable moon phases, especially on popular lakes. According to Jason [34]:

*“So many guys fish by the moon phase concerning muskie that it puts ten guys on the water now during a good moon phase to every one on a "non favorable" moon phase… You almost have to get in a bread line to fish a major spot during a prime moon phase on a major Minnesota, Wisconsin, or sometimes even Canadian, muskie fishery”*

The confounding effect on catch of variation in angler effort linked to anglers' perceptions of favorable fishing days cannot be discounted. We take as an ad hoc null hypothesis that the apparent lunar effect we detected was caused by the concentration of effort by anglers around specific lunar phases perceived to convey above-average angling success and *not* by any underlying biological effect of the moon on muskellunge behavior. Do our results, then, contain evidence that the apparent lunar effect is due, at least in part, to a muskellunge behavioral response to the lunar cycle? We submit that the following findings from our analysis support the alternative hypothesis that the observed lunar effects on catch are not due *entirely* to increased angler effort but reflect a biological response in the fish.

1. Variation among months in the strength of the lunar effect on catch suggests a life history component to the effect. Assuming that anglers do not abandon their affinity for fishing at certain times in the lunar cycle during June and October, the weaker lunar effect (Fig. 6) suggests that feeding activity of muskellunge in the circumspawning, and pre-winter period is less synchronized with the lunar cycle.
2. The shape of the response is suggestive. For example, there was a relatively smooth sinusoidal increase and decrease in catch numbers around the full and new moons for Ontario, Lake St. Clair, and elsewhere. We would *not* expect recreational anglers, who cannot fish every day, to behave this way. Rather we would expect peaks in angler effort on or very close to the full and new moons – the perceived best fishing days. This predicted behavior is akin to what we actually observed for effort at Mille Lacs and Lake Vermilion (Fig. 4). Fish, however, would entrain there cyclic behavior from the *continuous* lunar zeitgeber [1] – not from published moon phase tables.
3. The pattern in catch over the lunar cycle was different for daytime and nighttime caught fish. Daytime catch was equal during the full and new moon periods. Nighttime catch was higher during the full moon than during the new moon. For this result to be a consequence of angler effort, nighttime anglers would have to be strongly avoiding new moons periods relative to daytime anglers.
4. The catch of small muskellunge (<102 cm) was less strongly related to the lunar cycle than the catch of larger muskellunge (Fig. 5). Presumably, lunar variation in angler effort is independent of caught fish size. If effort *alone* were driving the catch pattern, then more effort during specific lunar periods would apply equally to all sizes of fish. Smaller fish have a faster metabolism than large fish and must feed more often [35], [36]. They may of necessity be less synchronized with the lunar cycle − a biological effect.
5. The strength of the lunar effect varied with latitude (Fig. 5). Is it likely that high latitude muskellunge anglers concentrate their effort around the new and full moon more than their more southerly confreres, or is a biological explanation more plausible (discussed further below)?
6. The lunar cycle explained the same amount of variation in catch for expert anglers and novice anglers, and lunar patterns were nearly identical (Fig. 8). We reason that many of the high-expertise anglers are professional guides and competitive tournament anglers that do not have the luxury of only fishing during the new and full moon. Occasional muskellunge anglers are unlikely to be sophisticated enough or fish often enough to focus their angling effort around the new and full moon. That the pattern in the catch of these two groups of anglers is nearly identical suggests an intrinsic rather than effort driven pattern in the catch through the lunar cycle.

### What Accounts for the Latitude Effect?

The sample-size-corrected effect of latitude on the lunar effect was unexpected, and we can only speculate on the underlying factors. One possibility is that overall angling pressure on high latitude muskellunge populations is lower than pressure on more southerly populations. Although the number of records from Ontario is similar to that of Wisconsin, Ontario anglers are likely spread over a much larger area (Fig. S1). If angling pressure disrupts lunar synchronization of muskellunge behavior, it could partly explain the difference in lunar influence between, for example, Ontario and Wisconsin or Ontario and Ohio. In relatively unexploited populations (where individual fish are presumably caught and released less often), there may be a more pure expression of the lunar effect on catch. To the degree that catch and release fishing pressure effects individual fitness, heritable change in behavior, including lunar asynchrony of feeding, may develop in hard-fished waters [37], [38].

### What is the Relative Effect of the Lunar Cycle?

Given how difficult it is to catch a muskellunge, any advantage that accrues to the moon-conscious angler is noteworthy. The maximum relative effect varied among fisheries. Overall, the effect was about 5%, but was higher (15–28%) for several popular muskellunge fisheries and for night fishing. When translated to the reduction in the time required to catch a musky (Table 2), our findings suggest that each muskellunge will be caught 2 to 5 h sooner by the lunar-phase-optimizing angler than by the angler choosing his fishing days at random. Expressed another way anglers “fish the moon”, the “Fish of 10,000 Casts” [24] becomes the fish of about 9,500 casts.

### What is the Underlying Explanation for the Lunar Effect?

A variety of explanations for the lunar effect has been proposed. Knight [4] invoked “inland tides” in barometric pressure and gravitational forces. Using reasoning too subtle for us to grasp, he developed his Solunar Tables, which are still published (http://www.solunarforecast.com). As far as we can discern, however, Knight [4], [39] never articulated a causal biological mechanism for the Solunar phenomenon. An oft-cited explanation for a lunar effect is that the amount of lunar illumination influences nocturnal feeding activity, which is inversely related to daytime catchability. This idea was well expressed by Stevenson and Miller [40]:

*“Feeding at night during the full moon is plausibly much easier that it is during the new moon,…as the extra light available would make it easier to spot potential prey. It is likely that [fish] are hungrier during the day following a darker night, therefore biting with higher intensity, resulting in larger catches for recreational fishers.”*

Our finding of nearly equal peaks in daytime catch during the full and new moons suggests that lunar illumination per se does not account for variation in the muskellunge catch.

Lunar synchronization must convey, or have conveyed in the past, some fitness advantage to the fish [41]. Presumably, synchronization somehow increases energy intake by muskellunge (since predation risk is probably not a strong selective force for adult muskellunge). Marine pelagic predators track prey associated with the deep scattering layer (DSL). Fish such as tuna and sharks that are adapted to feed at variable depths move deeper during periods of the full moon following the DSL where they are less vulnerable to anglers [14]. In an inversion of this scenario, diurnal, surface-feeding istiophorid billfishes were more vulnerable to angling during the full moon period because the reduction in available prey (which have dived with the DSL), makes fish desperate for food [14]. Physiological adaptation for feeding at depth probably does not apply to the muskellunge, which likely evolved as a river species and has relatively recently invaded the deeper lentic environment [19]. Lunar gravitation has been suggested as a cue to fish behavior [42], including in pike [16]. The biological mechanisms that link muskellunge behavior to the moon remain a mystery, however.

### Are There Other Factors That Influence Catchability?

The lunar cycle explains a significant part of the variation in catch over time during the angling season. Clearly, the moon does not *determine* muskellunge-fishing success since the number of fish caught during even the “worst” part of the lunar cycle never approaches zero (Fig. 3). Obviously, more than the moon is involved in the chain of events that leads to an individual muskellunge striking an angler's bait. After all, few anglers would gainsay that weather accounts for much of the variation in fish feeding. Another intriguing possibility is the role of variation in behavior among individual fish in a particular water body, including, presumably, response to lunar influence. Recent research [44], [43] has shown that multiple behavioral types may exist in pike populations in which individuals with different foraging strategies have equal fitness. Similar research on muskellunge may reveal that some individual fish are more synchronized with the lunar cycle than others.

## Conclusion

Hanson et al. [20] stated in 2008 that “there was no conclusive scientific study to support claims that temperate sport fish behavior varies… in response to changing lunar periods in a manner that increases the likelihood of capturing fish via recreational angling.” Alas, because we have limited effort data, our study is not conclusive. We have shown, however, that angler catch of muskellunge is strongly related to the lunar cycle. This occurs, at least in part, because muskellunge feeding behavior is synchronized with the lunar cycle – that the observed patterns in angler catch do not simply reflect biased (non-random) angler effort. We have noted several sources of variation in the lunar pattern including, latitude, time of day, month, and fish size that seem to us evidence of biological mediation of the lunar effect.

## Supporting Information

Figure S1 **Location of muskellunge catch records.** Coordinates for the location of the water body for Canadian records and for the centroid of the county for U.S. records.
(DOCX)

Figure S2 **Simulated patterns for periodic regression models with two lunar predictors.** For each simulation, *a* was set at 4; *x_1_ and x_2_ were* set at 4. The lunar cycle starts at 0001 h on the morning after the full moon.
(DOCX)

Figure S3 **Effect of the number of model predictors on the model strength for a randomly selected sample of lunar days.** Each bar represents the mean *R^2^* value (±95% CI) for 100 runs of the *model y = a+xcosine2θ*, where *y* = number of fish caught and *θ* is the angular equivalent of lunar day. Models were run on a randomly-selected sample of 50,000 lunar days from 1 to 29 (on which a simulated fish was “caught”).
(DOCX)

Figure S4 **Regression diagnostic plots for the effect of lunar day on all fish caught in daytime and on angler effort at Lake Vermilion.** Normal probability plots (top row) show that residuals are approximately normally distributed. Residual plots (bottom row) show that a linear model is appropriate and that residuals are homoscadistic. Regression statistics give in Table 1.
(DOCX)

Figure S5 **Total number of fish caught by day of the week from the Muskies Inc. dataset (Plot A).** Total number of anglers by day of the week from state Department of Natural Resources creel data. Data combined for Wisconsin, Lake Vermilion, and Mille Lacs (Plot B). Number of occurrences of selected days of the week by lunar day for the period of record of the Muskellunge Inc. data (1970–2013; Plot C). The expected number of occurrences of each lunar day during the period of record absent a lunar effect is 78.4.
(DOCX)

Figure S6 **Percent of muskellunge catch by month and latitude.** Each color sums to 100%.
(DOCX)

Figure S7 **LOESS and periodic regression results for catch records for selected locations.** Dashed regression lines are for the best two-predictor periodic regression model (regression statistics in Table 1). Solid lines are the LOESS fit with sampling proportion  = 0.3.
(DOCX)
