# Supplementary material for: Muskie Lunacy: Does the Lunar Cycle Influence Angler Catch of Muskellunge (Esox masquinongy)?
Source: PLoS One. 2014 May 28;9(5):e98046. doi: 10.1371/journal.pone.0098046 (PMC4037224; doi:10.1371/journal.pone.0098046)
Supplement: Figure S1 — Location of muskellunge catch records. Coordinates for the location of the water body for Canadian records and for the centroid of the county for U.S. records. (DOCX) [file pone.0098046.s001.docx]

**Figure S1 Location of muskellunge catch records. C**oordinates for the location of the water body for Canadian records and for the centroid of the county for U.S. records.

**
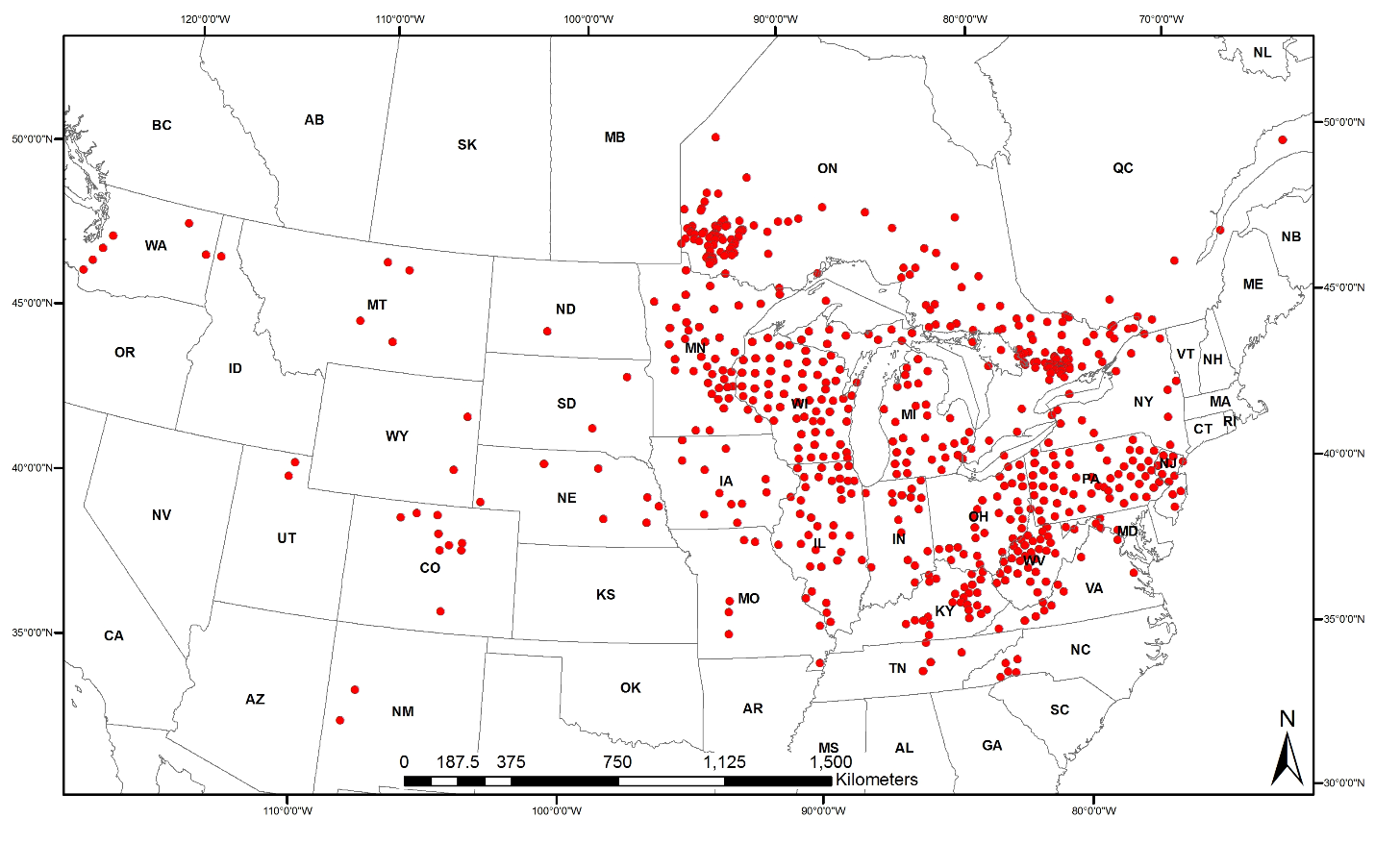
**
