# Supplementary material for: Muskie Lunacy: Does the Lunar Cycle Influence Angler Catch of Muskellunge (Esox masquinongy)?
Source: PLoS One. 2014 May 28;9(5):e98046. doi: 10.1371/journal.pone.0098046 (PMC4037224; doi:10.1371/journal.pone.0098046)
Supplement: Figure S2 — Simulated patterns for periodic regression models with two lunar predictors. For each simulation, a was set at 4; x1 and x2 were set at 4. The lunar cycle starts at 0001 h on the morning after the full moon. (DOCX) [file pone.0098046.s002.docx]

**Figure S2 Simulated patterns for periodic regression models with two lunar predictors.** For each simulation, *a* was set at 4; *x_1_ and x_2_ were* set at 4. The lunar cycle starts at 0001 h on the morning after the full moon.

**
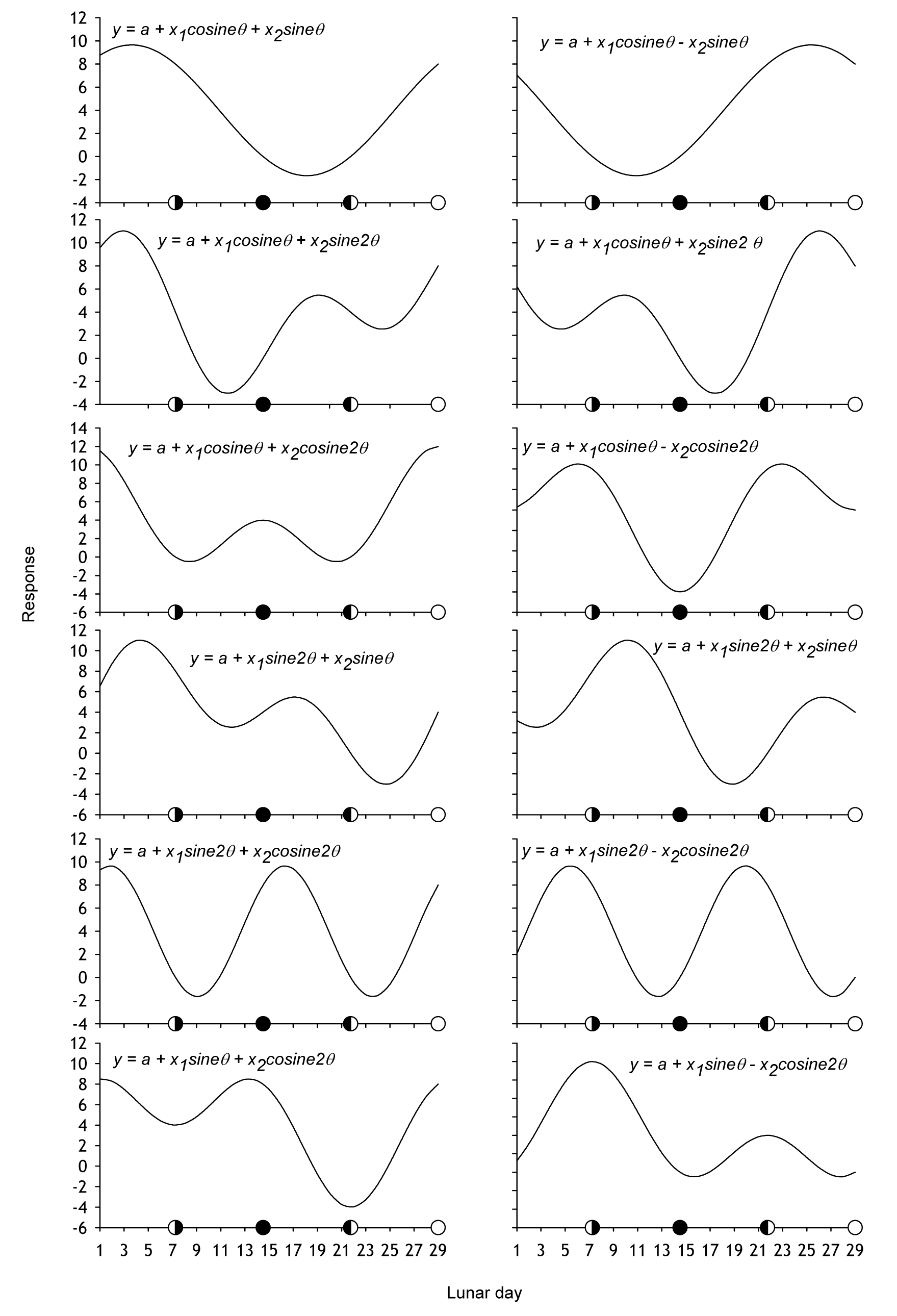
**
