# Supplementary material for: Muskie Lunacy: Does the Lunar Cycle Influence Angler Catch of Muskellunge (Esox masquinongy)?
Source: PLoS One. 2014 May 28;9(5):e98046. doi: 10.1371/journal.pone.0098046 (PMC4037224; doi:10.1371/journal.pone.0098046)
Supplement: Figure S3 — Effect of the number of model predictors on the model strength for a randomly selected sample of lunar days. Each bar represents the mean R2 value (±95% CI) for 100 runs of the model y = a+xcosine2θ, where y = number of fish caught and θ is the angular equivalent of lunar day. Models were run on a randomly-selected sample of 50,000 lunar days from 1 to 29 (on which a simulated fish was “caught”). (DOCX) [file pone.0098046.s003.docx]

**Figure S3 Effect of the number of model predictors on the model strength for a randomly selected sample of lunar days.** Each bar represents the mean *R^2^* value (+95% CI) for 100 runs of the *model y = a + xcosine2θ,* where *y* = number of fish caught and *θ* is the angular equivalent of lunar day. Models were run on a randomly-selected sample of 50,000 lunar days from 1 to 29 (on which a simulated fish was “caught”).

**
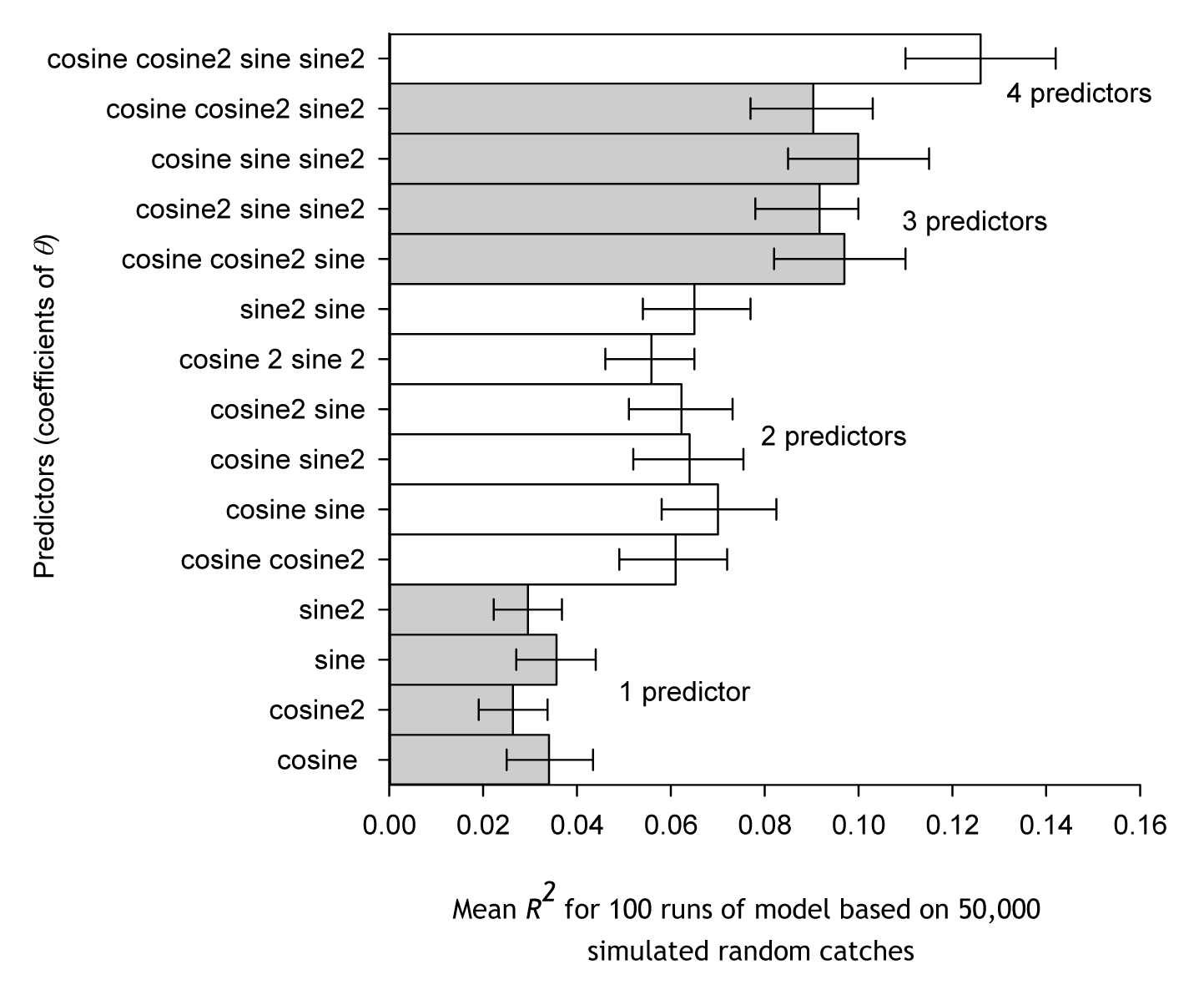
**
