# Supplementary material for: Muskie Lunacy: Does the Lunar Cycle Influence Angler Catch of Muskellunge (Esox masquinongy)?
Source: PLoS One. 2014 May 28;9(5):e98046. doi: 10.1371/journal.pone.0098046 (PMC4037224; doi:10.1371/journal.pone.0098046)
Supplement: Figure S4 — Regression diagnostic plots for the effect of lunar day on all fish caught in daytime and on angler effort at Lake Vermilion. Normal probability plots (top row) show that residuals are approximately normally distributed. Residual plots (bottom row) show that a linear model is appropriate and that residuals are homoscadistic. Regression statistics give in Table 1. (DOCX) [file pone.0098046.s004.docx]

**Figure S4 Regression diagnostic plots for the effect of lunar day on all fish caught in daytime and on angler effort at Lake Vermilion.** Normal probability plots (top row) show that residuals are approximately normally distributed. Residual plots (bottom row) show that a linear model is appropriate and that residuals are homoscadistic. Regression statistics give in Table 1.

**
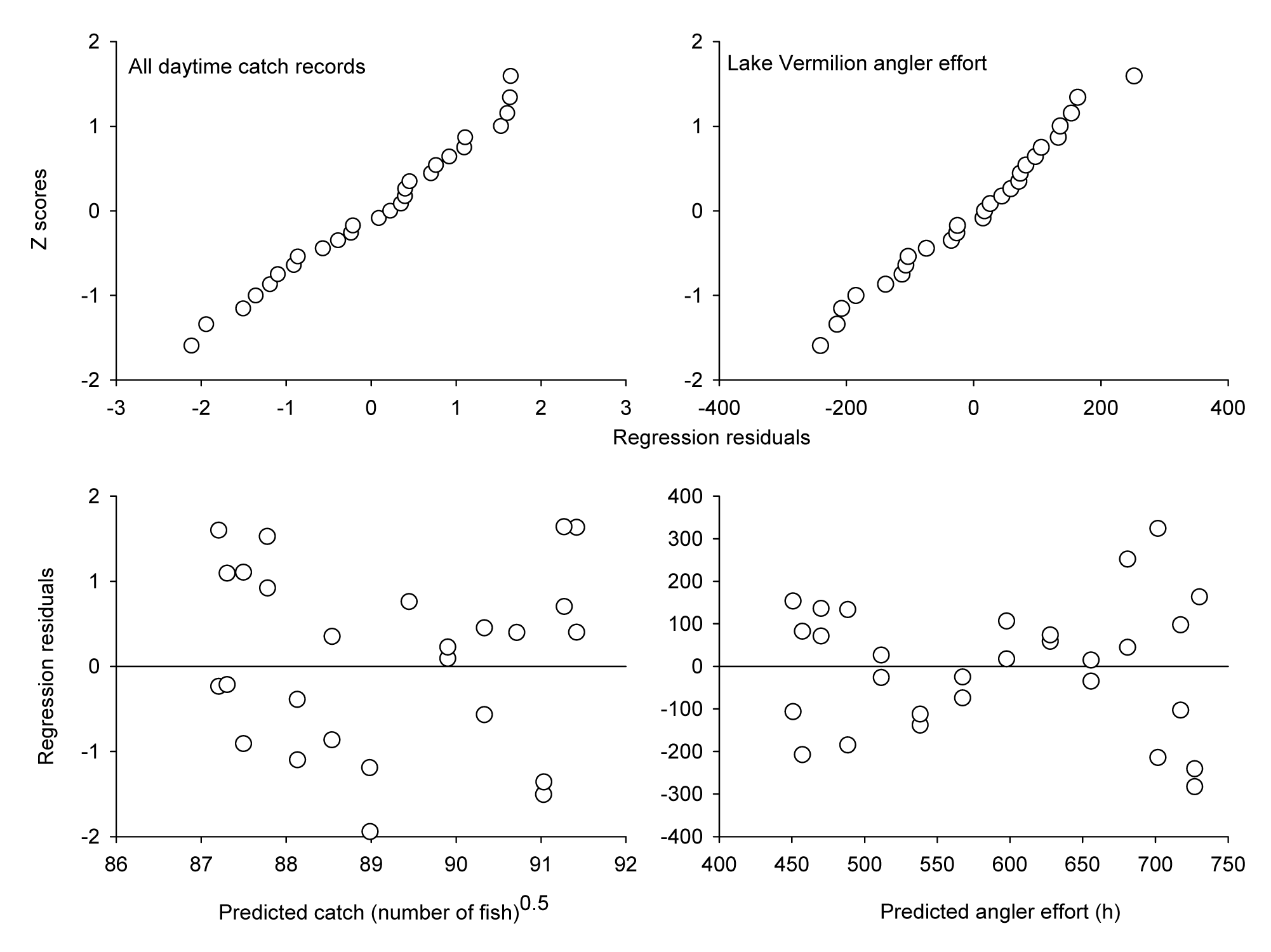
**
