# Supplementary material for: Muskie Lunacy: Does the Lunar Cycle Influence Angler Catch of Muskellunge (Esox masquinongy)?
Source: PLoS One. 2014 May 28;9(5):e98046. doi: 10.1371/journal.pone.0098046 (PMC4037224; doi:10.1371/journal.pone.0098046)
Supplement: Figure S5 — Total number of fish caught by day of the week from the Muskies Inc. dataset (Plot A). Total number of anglers by day of the week from state Department of Natural Resources creel data. Data combined for Wisconsin, Lake Vermilion, and Mille Lacs (Plot B). Number of occurrences of selected days of the week by lunar day for the period of record of the Muskellunge Inc. data (1970–2013; Plot C). The expected number of occurrences of each lunar day during the period of record absent a lunar effect is 78.4. (DOCX) [file pone.0098046.s005.docx]

**Figure S5 Total number of fish caught by day of the week from the Muskies Inc. dataset (Plot A).** Total number of anglers by day of the week from state Department of Natural Resources creel data. Data combined for Wisconsin, Lake Vermilion, and Mille Lacs (Plot B). Number of occurrences of selected days of the week by lunar day for the period of record of the Muskellunge Inc. data (1970−2013; Plot C). The expected number of occurrences of each lunar day during the period of record absent a lunar effect is 78.4

**
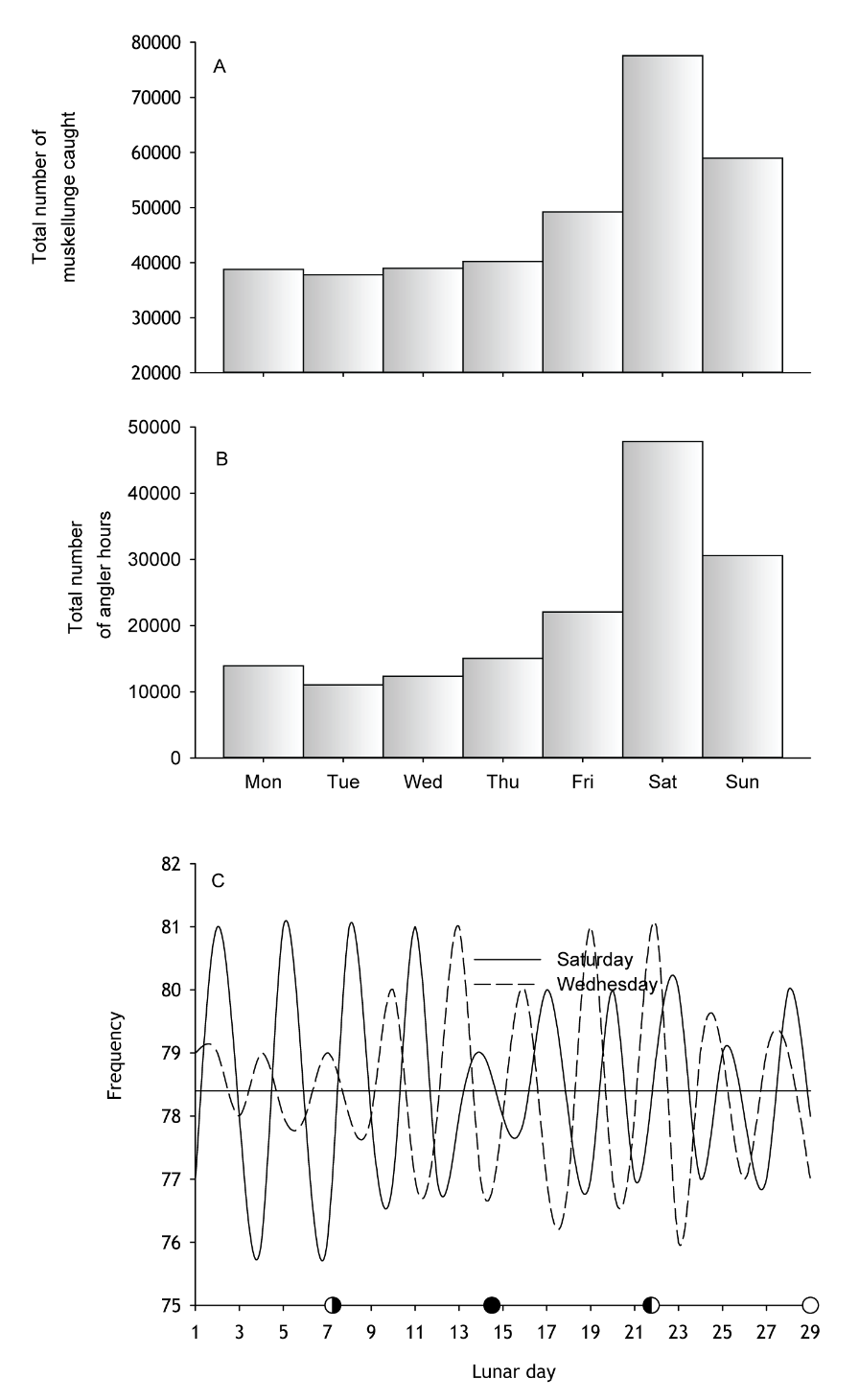
**
