# Supplementary figures and images for: Muskie Lunacy: Does the Lunar Cycle Influence Angler Catch of Muskellunge (Esox masquinongy)?
Source: PLoS One. 2014 May 28;9(5):e98046. doi: 10.1371/journal.pone.0098046 (PMC4037224; doi:10.1371/journal.pone.0098046)

**Figure S6 Percent of muskellunge catch by month and latitude.** Each color sums to 100%

**
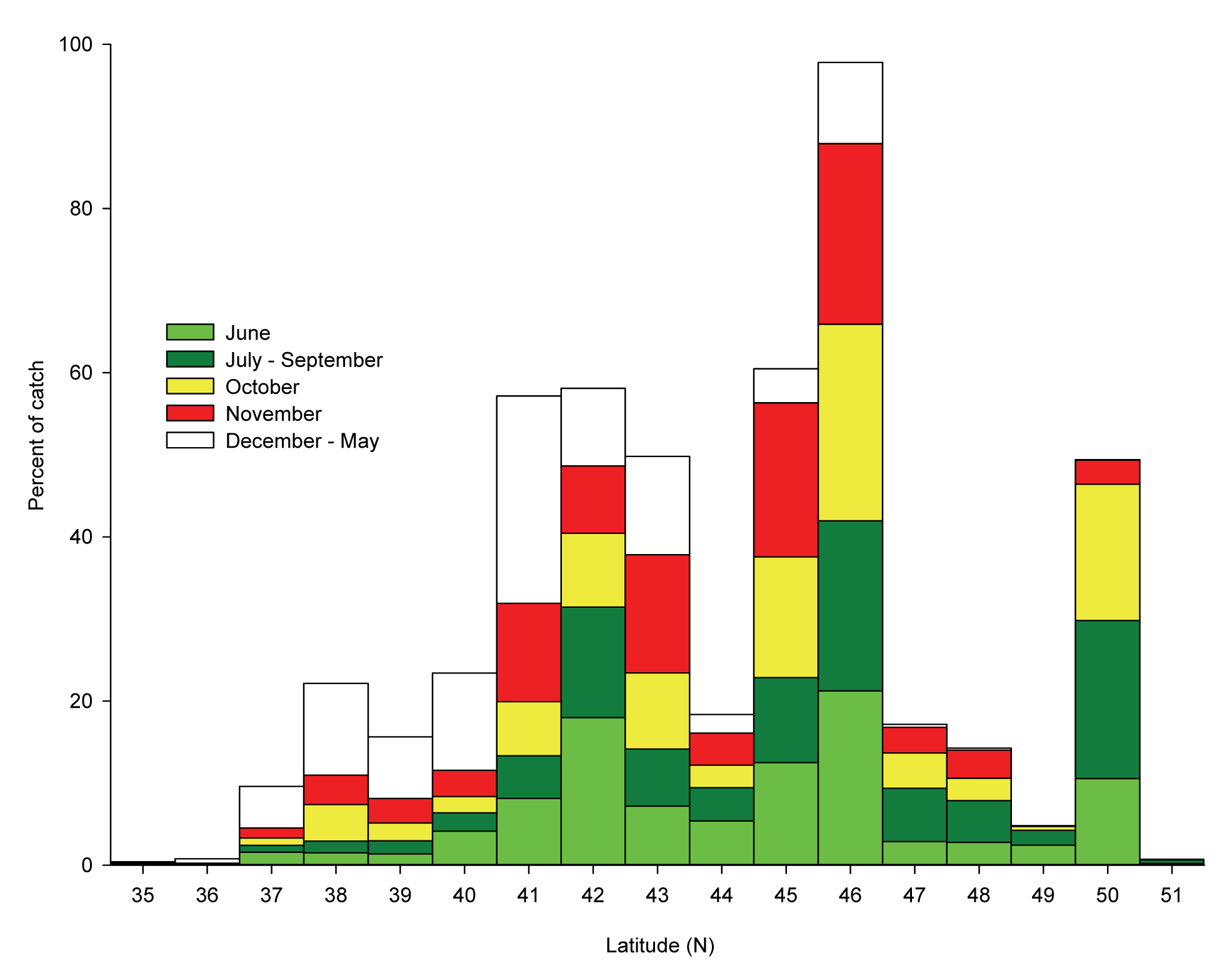
**

Supplement: Figure S6 — Percent of muskellunge catch by month and latitude. Each color sums to 100%. (DOCX) [file pone.0098046.s006.docx]
