# Supplementary material for: Muskie Lunacy: Does the Lunar Cycle Influence Angler Catch of Muskellunge (Esox masquinongy)?
Source: PLoS One. 2014 May 28;9(5):e98046. doi: 10.1371/journal.pone.0098046 (PMC4037224; doi:10.1371/journal.pone.0098046)

**Figure S7 LOESS and periodic regression results for catch records for selected locations.** Dashed regression lines are for the best two-predictor periodic regression model (regression statistics in Table 1). Solid lines are the LOESS fit with sampling proportion =0.3.


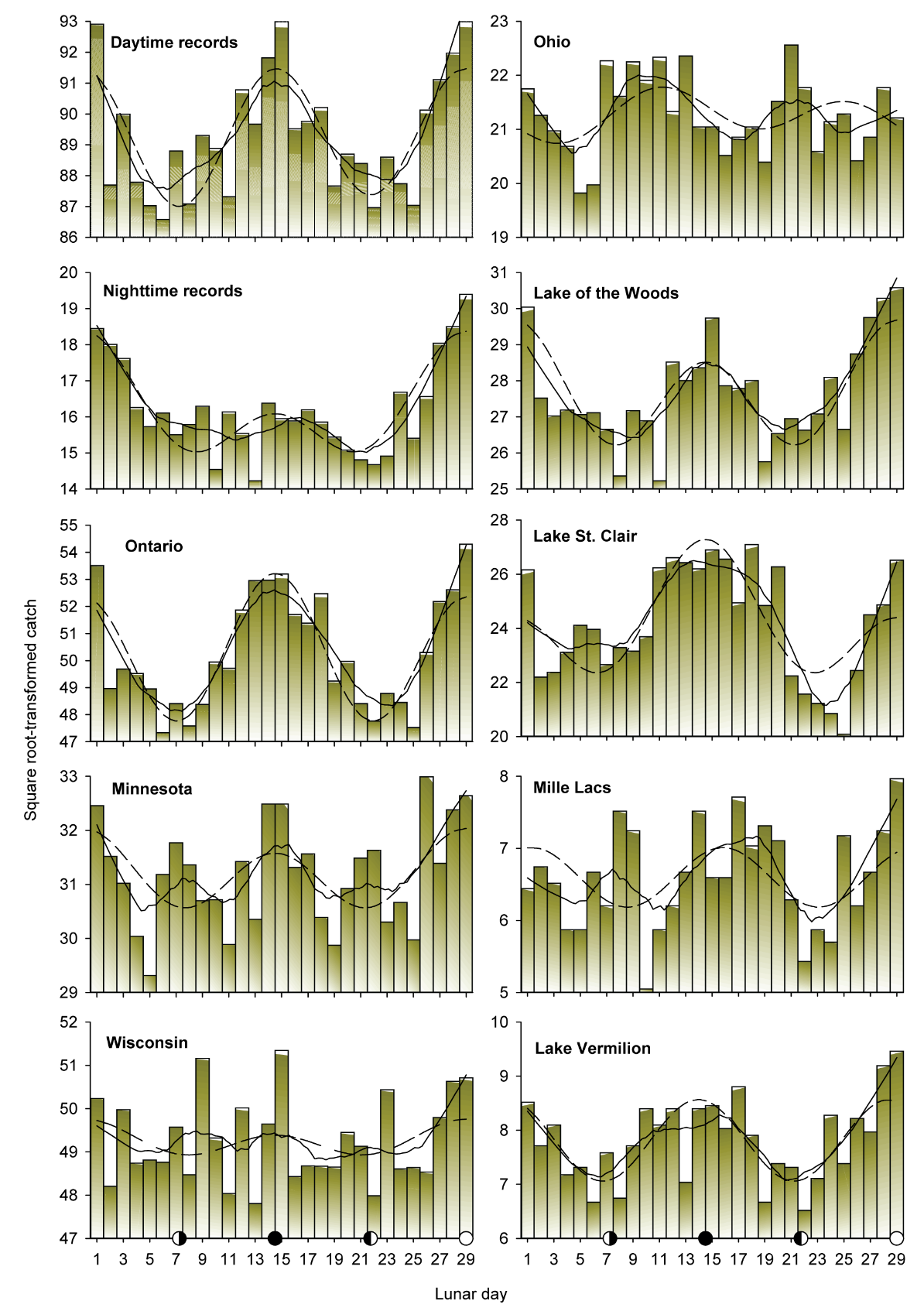

Supplement: Figure S7 — LOESS and periodic regression results for catch records for selected locations. Dashed regression lines are for the best two-predictor periodic regression model (regression statistics in Table 1). Solid lines are the LOESS fit with sampling proportion = 0.3. (DOCX) [file pone.0098046.s007.docx]
